# Clinically informative microRNAs for SARS-CoV-2 infection

**DOI:** 10.2217/epi-2023-0179

**Published:** 2023-09-04

**Authors:** Sercan Ergün, Ramamoorthy Sankaranarayanan, Nina Petrović

**Affiliations:** ^1^Department of Medical Biology, Faculty of Medicine, Ondokuz Mayis University, Samsun, Turkey; ^2^Department of Multidisciplinary Molecular Medicine, Institute of Graduate Studies, Ondokuz Mayis University, Samsun, Turkey; ^3^Department of Plant Sciences, School of Biological Sciences, Madurai Kamaraj University, Tamil Nadu, India; ^4^Laboratory for Radiobiology & Molecular Genetics, Department of Health & Environment, ‘VINČA’ Institute of Nuclear Sciences – National Institute of the Republic of Serbia, University of Belgrade, Mike Petrovića Alasa 12–14, Belgrade, 11001, Serbia; ^5^Department of Experimental Oncology, Institute for Oncology & Radiology of Serbia, Pasterova 14, Belgrade, 11000, Serbia

**Keywords:** COVID-19, diagnosis, microRNA, SARS-CoV-2, transcriptional regulation, viral infection

## Abstract

COVID-19 is a viral respiratory infection induced by the newly discovered coronavirus SARS-CoV-2. miRNA is an example of a strong and direct regulator of a gene’s transcriptional activity. The interaction between miRNAs and their target molecules is responsible for homeostasis. Virus-derived and host-derived miRNAs are involved in the activity of hiding from immune system cells, inducing the inflammatory reaction through interplay with associated genes, during SARS-COV-2 infection. Interest in miRNAs has raised the comprehension of the machinery and pathophysiology of SARS-COV-2 infection. In this review, the effects and biological roles of miRNAs on SARS-CoV-2 pathogenicity and life cycle are described. The therapeutic potential of miRNAs against SARS-CoV-2 infection are also mentioned.

## Tweetable abstract

Small regulatory nucleic acids, miRNAs, may provide diagnostic and therapeutic potential for COVID-19.

COVID-19 is a respiratory infection induced by the coronavirus SARS-CoV-2. The COVID-19 pandemic has brought substantial health-related issues for all human beings. Even though various attempts have been made to elucidate the pathways involved during the coronavirus proliferation, remarkable gaps remain in our comprehension of COVID-19’s pathogenic development.

miRNAs are noncoding, tiny RNAs having crucial functions in varied biological actions via suppressing the transcriptional activities of genes. They are composed of RNA molecules 18–24 nucleotides in length. Latterly, miRNAs have been considered as significant regulators of viral infections. Not only viral miRNAs but also host miRNAs can have valuable diagnostic and therapeutic potential for SARS-CoV-2 infection. In the light of this information, clinically informative miRNAs for SARS-CoV-2 infection are analyzed under five subtopics in this review: miRNAs contributing to susceptibility to SARS-CoV-2 infection (in other words; miRNAs making SARS-CoV-2 infection easier); miRNAs having therapeutic potential against SARS-CoV-2 infection; miRNAs contributing to resistance to SARS-CoV-2 infection (in other words; miRNAs making SARS-CoV-2 infection more difficult); miRNAs as potential indicators of worse clinical outcome in SARS-CoV-2-infected individuals; and miRNAs as potential indicators that disease would be less harmful for SARS-CoV-2-infected individuals.

## miRNAs contributing to susceptibility to SARS-CoV-2 infection

A host miRNA could interact with the virus’s infectious cycle in two important ways: it may bind to the virus genome by complementary base pairing and prevent the virus translation, or it may stabilize the genome and even increase viral replication [1]. According to this standpoint, viruses may evolve to utilize the host miRNAs which are favorable, while mutating regions where the host translation-suppressing miRNAs are complementary. One such study accessed the SARS-CoV-2 variants in UTRs and their impacts on miRNA crosstalk, and showed how miR-34b-5p, miR-3664-5p, miR-9-5p and miR4701-3p (along with RNA-binding proteins) interact with 3′ UTR variants of SARS-CoV-2 [2]. Moreover, miR-9 is sequestered by the human coronavirus OC43 and hence NF-κB is overexpressed [3]. Interestingly, Das *et al*. reported that miR-27b is specific to the Indian population, even binding to the mutant domain of the Indian strain of SARS-CoV-2, and has antiviral effects [4]. As the virus mutation rate is higher, such investigations are important to understand the evolution and hence the miRNA mediations.

Serum levels of hsa-miR-29a-3p, hsa-miR-31-3p and hsa-miR-126-3p are reduced with increased severity of COVID-19, and the relative expression levels of *COL5A3*, *ZMYM5* and *CAMSAP1* are also increased with increased severity of COVID-19. However, hsa-miR-17-3p behaves in the opposite way: serum levels of hsa-miR-17-3p were found to rise with increased disease severity, and the relative expression of *DICER1* is increased. In the same study, the authors noted that during hospitalization, the aforesaid miRNAs’ relative expression levels tended to become normal [5]. Interestingly, hsa-miR-29a-3p has five direct binding sites on ORF1ab, the spike nucleocapsid of the SARS-CoV-2 genome. Computation analysis showed that the miRNA–virus RNA binding free energy was less than -30 kcal/mol and had a perfect seed match [6]. These results suggest that the circulatory levels of hsa-miR-31-3p, hsa-miR-126-3p, hsa-miR-29a-3p and hsa-miR-17-3p would be helpful to diagnose the prognosis of COVID-19 patients.

Differential miRNA expression in COVID-19 patients provides insights into patient management. In one study, analysis of plasma samples from diseased and healthy controls, followed by sequencing studies, revealed that 50 miRNAs showed significantly altered expression patterns; among them, miR-3125, miR-31-5p and miR-4742-3p were overexpressed while miR-766-3p, miR-1275, miR-3617-5p and miR-500b-3p were downregulated significantly. In the same study, the authors found that three miRNA signatures, consisting of miR-423-5p, miR-195-5p and miR-23a-3p, classified SARS-CoV-2 patients with great accuracy [7]. Interestingly, miR-195’s disease association with lung neoplasia can be predicted using an *“anti-noise based computational model for predicting potential miRNA-disease associations”* [8]. Using a combination of miRNAs for diagnostic purposes is not a new approach and is also known as the universal screening test. It has been proposed that many diseases and cancers could be diagnosed by such a universal screening test [9–11]. Moreover, the search for disease associations of miRNAs is not yet over. Dozens of methods have been proposed using different computational approaches [12–15]. However, the applications of these methods for SARS-CoV-2 are limited and more such studies in the future predict propitious miRNA candidates instead of the innocent bystanders, to address the infection better.

## miRNAs having therapeutic potential against SARS-CoV-2 infection

The first miRNA was discovered in 1993, and miRNA of viral origin was proposed approximately a decade later [16,17]. In 2007, Watanabe *et al.* assessed 470 available human miRNA target sites against 228 human-infecting virus genomes. In this comprehensive study, they showed that out of 470 miRNAs, 388 were putatively targeting the virus genome. Many of them target multiple viruses [18]. To date, 2654 miRNAs of human origin have been listed in the miRBase (https://mirbase.org/). Plenty of them have a significant involvement in host–virus interactions and are more likely to have therapeutic potential [19]. Having one or more miRNA target sites on the 3′ UTR of the virus genome is a potential sign of virus translation suppression [20].

Many computational approaches have revealed the presence of putative host miRNA binding sites on the SARS-CoV-2 genome. miRNAs like hsa-miR-25-5p, hsa-miR-9-5p, hsa-miR-34b-5p, hsa-miR-196b-5p, hsa-miR-1293, hsa-miR-2116-5p, hsa-miR-323b-5p and hsa-miR-4659b-3p have been shown to have a complementary region on the SARS-CoV-2 genome [21]. As the positive-strand RNA virus genome closely mimics the cellular mRNA, host miRNAs interact more readily with the virus genome and regulate the virus infectious cycle, hence having more therapeutic efficacy. Such direct host miRNA interactions affecting viral pathogenesis have been studied in dozens of viruses [1]. Another similar investigation suggested the high-confidence target sites of miR-6089-5p, miR-4763-3p, miR-103a-3p, miR-1207-5p and miR-6821-5p on the SARS-CoV-2 genome [22]. In another study, Sardar *et al.* claimed that 42 miRNAs (out of 186, targeting the SARS-CoV-2 genome) were conserved and had complementary sequences in different geographical isolates of the SARS-CoV-2 genome; miRNAs such as hsa-miR-138-5p, hsa-miR-761, hsa-miR-622, hsa-miR-15b-5p, miR-A3r, hsa-miR-18a-5p, miR-A2r and miR-B1r had three or more targets on the SARS-CoV-2 genome, preferably in the S protein and ORF1ab genes. The authors also pointed out that three antiviral miRNAs (miR-125a, miR-198 and miR-23b) had pivotal roles in respiratory diseases [23].

**Figure 1.**
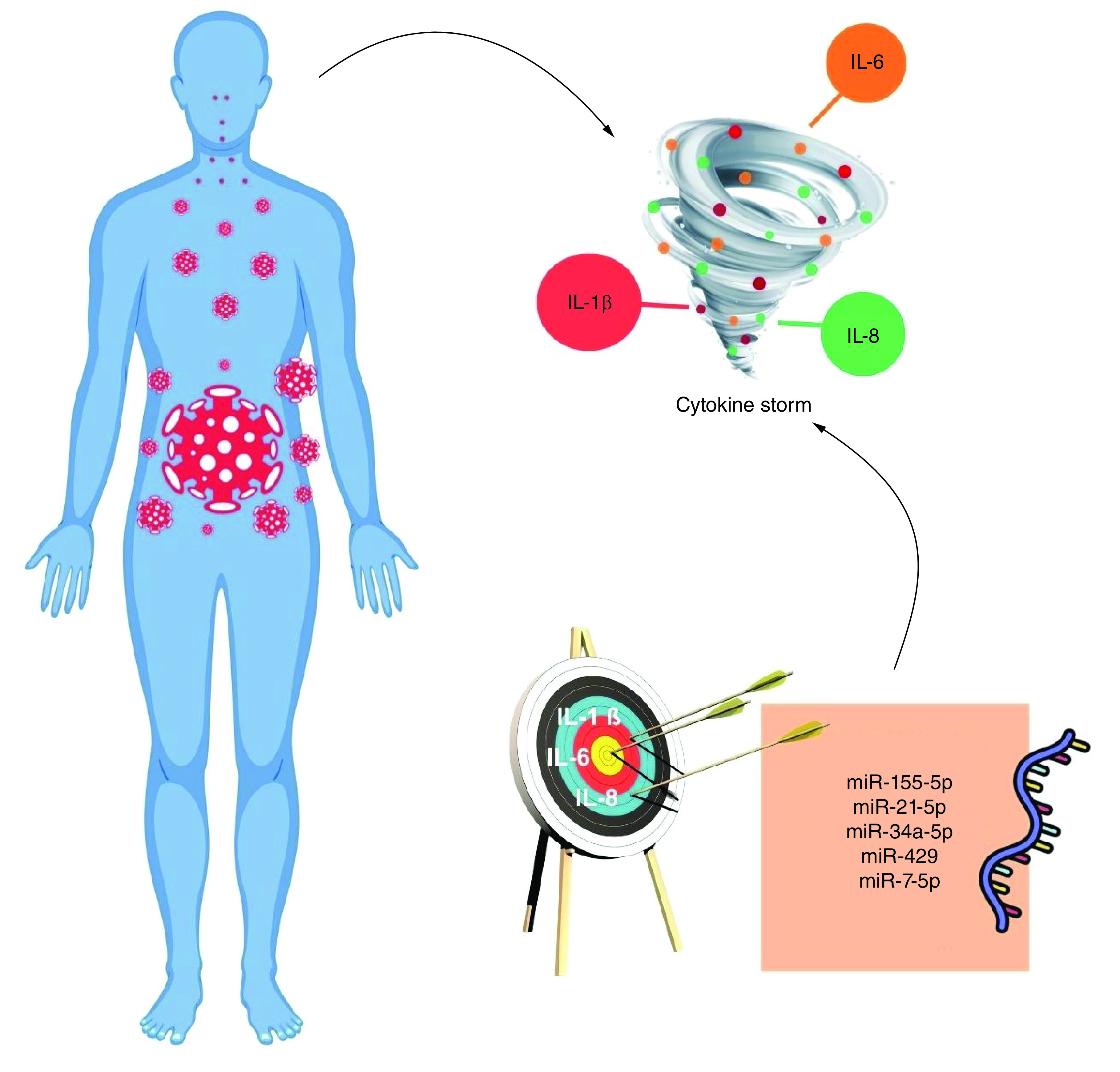
The role of microRNAs in the cytokine storm during COVID-19.

The COVID-19-associated cytokine storm is the ‘dark age’ of the infection because it results in a high mortality rate. It is activated by several cytokines, chemokines and growth factors [24]. Among them, IL-1β, IL6 and IL8 are the most notable. Clinical trials aiming to target the mentioned interleukins are proposed (NCT04603742, NCT04381052 and NCT04247226) (https://clinicaltrials.gov/). Gasparello *et al.* listed 89 miRNAs that were targeting the mentioned interleukins; among them, miR-21-5p, miR-155-5p, miR-34a-5p, miR-429 and miR-7-5p targeted all three interleukins; 23 miRNAs targeted two of the interleukins; and the rest targeted any one of the interleukins (Figure 1) [25].

Hu *et al.* showed that many human miRNAs share the complementary binding region with the SARS-CoV-2 genome compared with SARS-CoV [26]. Kyoto Encyclopedia of Genes and Genomes pathway analysis and gene ontology studies by Arisan *et al.* revealed that seven miRNAs (miR-8066, miR-3611, miR-5197-3p, miR-3934-3p, miR-1307-3p, miR-3691 and miR-1468-5p) had potential therapeutic value. Notably, miR-5197-3p, miR-4778-3p and miR-6864-5p showed significant binding affinity on SARS-CoV-2 guide RNA, providing hope for therapeutic strategies. miR-5197-3p and miR-8066 were found to be involved in mucin-type O-glycan biosynthesis. Kyoto Encyclopedia of Genes and Genomes pathway analysis showed the association of the biosynthesis pathway with many viral infections like human T-cell leukemia virus type 1, Ebola virus and influenza virus [27].

## miRNAs contributing to resistance to SARS-CoV-2 infection

Host miRNAs can possess a well-organized function in inhibiting viral attack of the host system via arresting target pathways required for viral diffusion and other important pathways needed for virus penetration and replication. For example, miR-155 was shown to be expressed to suppress Dengue virus replication by stimulating antiviral interferon reactions via heme oxygenase-1 induction and downregulating the similar BACH1 pathway [28]. Moreover, infrequent potential loss-of-function forms of X-chromosomal Toll-like receptor 7 inducing immune system-related failures in type I and II interferon generation were recently identified in four young male patients with serious COVID-19 [29]. The reactions to type I and II interferons have been implicated in the induction of an early immune response to remove SARS-CoV-2 and suppress the progression of COVID-19, so the presence of miR-155 overexpression may provide resistance against SARS-CoV-2 infection through inducing type I and II interferon production or may provide an informative signal as a potential biomarker for immune system activation against SARS-CoV-2 [29]. Moreover, one study showed that various miRNAs might have a direct function in modulating viral infectivity through constructing miRNA profiling data of both A549 (low SARS-CoV-2 infectivity) and Huh7 (high SARS-CoV-2 infectivity). The study team detected that miR-23a, miR-23b, miR-29a and miR-29c were upregulated in the cell lines showing low SARS-CoV-2 infectivity (primary lung fibroblasts and A549), whereas they were downregulated in cell lines with high SARS-CoV-2 infectivity (Huh7 and Calu-3) [30]. Thus it can be inferred that miR-23a, miR-23b, miR-29a and miR-29c overexpression made the cells more resistant to SARS-CoV-2 infection. Moreover, according to a functional enrichment study, miR-34a-5p, miR-26a-5p and miR-29b-3p were defined as modulators of mRNA targets included in viral diseases. When compared with the controls (n = 10), downregulation of the defined miRNAs was detected in the COVID-19 patients’ lung biopsies (n = 9) (p < 0.01–0.0001). Altogether, these findings demonstrated that miR-34a-5p, miR-26a-5p and miR-29b-3p upregulation provides a signal for resistance to SARS-CoV-2 infection [31]. In another study, five miRNAs (hsa-miR-196a-1-3p, hsa-miR-16-5p, hsa-miR-15b-5p, hsa-miR-15a-5p and hsa-miR-195-5p) were observed to frequently bind to SARS-CoV-2, Middle East respiratory syndrome coronavirus and SARS-CoV [32]. The expression profiles of these miRNAs were analyzed in hamster lung tissues infected by SARS-CoV-2. Overexpressed hsa-miR-195-5p triggers apoptosis through stimulating cell cycle arrest and inhibits uncontrolled reproduction of the infected cells as the host immune reaction, whereas downregulation of hsa-miR-15b-5p can facilitate SARS-CoV-2 to escape the host immune defense mechanism by suppressing apoptosis and stimulating the reproduction of infected cells. Thus hsa-miR-195-5p downregulation and hsa-miR-15b-5p overexpression provide a signal for resistance against SARS-CoV-2 infection [32].

Some studies have especially focused on the regulation of SARS-CoV-2 entry points on infected cells via miRNAs. One study presented that miR-200c downregulation was necessary for SARS-CoV-2 binding to ACE2 in cardiomyocytes. The upregulation of miR-200c may decrease protein and mRNA levels of ACE2, and the particular pathway specified that miR-200c can target the 3′ UTR of *ACE2* mRNA [33]. Additional research determined that miR-98-5p could suppress transcriptional activity of *TMPRSS2*, another SARS-CoV-2 entry point, through targeting its 3′ UTR in human endothelial cells and so reduce SARS-CoV-2 infectivity [34]. Furthermore, Nersisyan *et al.* performed an *in silico* investigation to show that JARID1B may modulate *TMPRSS2* and *ACE2* through transcriptional suppression of miR-200/141 and miR-125a/let-7e, which immediately bind the 3′ UTRs of those two receptors, and that JARID1B activity was essential for those two receptors. Therefore, these miRNAs are significant for the activity of TMPRSS2 and ACE2 [35]. Moreover, miR-125b-5p may be the principal suppressor of ACE2 in lung adenocarcinoma. When *ACE2* is downregulated, IL-6 in the Toll-like receptor mechanism may trigger the immunity like a downstream actor. In particular, miR-125b-5p has a reverse-directed expression tendency with ACE2 [36]. Furthermore, *ACE2* is inhibited via binding of its 3′ UTR by hsa-miR-23b-5p and hsa-miR-769-5p [37]. Accordingly, miR-125b-5p was considered as a predicted upstream suppressor of *ACE2*.

Various particular human miRNAs have been shown to bind and suppress viral genes functioning in replication, translation and protein synthesis. Many studies have emphasized miRNAs directly targeting the SARS-CoV-2 genome and its products to investigate the effects of them on infectivity. It has been observed that the positive-sense and single-stranded RNA structure of the SARS-CoV-2 genome makes it sensitive to be targeted and regulated via host miRNAs [38]. Upon this finding, miR-8075, miR-7851-3p and miR-298 were identified to predict binding with elevated potential to the SARS-CoV-2 genome’s 5′ UTR [30]. The multibasic cleavage region is a specific site in the SARS-CoV-2 S protein which is not present in SARS-CoV or in pangolin and bat coronaviruses[30]. miR-4707-3p, miR-151b-5p and miR-151a-5p were shown to bind this site in the genome of SARS-CoV-2, and analysis of the secondary structure in that site supports availability for miRNAs targeting [30]. The upregulation of miR-151b-5p and miR-151a-5p in lung primary fibroblasts and lungs suggests a probable inclusion of those miRNAs in the stabilities of SARS-CoV-2 RNA and spike protein. Furthermore, Nsp12, Nsp8 and Nsp7 have newly been defined as crucial proteins in the replication of viruses and are bound by remdesivir, an investigational therapeutic agent. In one study, miR-3149, miR-320b and miR-320a-3p were estimated to target the Nsp12 and Nsp8 genomic sites of the replicase polyprotein 1ab (PP1ab), a substructure that is crucial in replication and transcription of viral RNAs [30]. Additionally, miR-29a/29b-1-5p/29c/3p/23a/23b are estimated to target many different sites of the genome of SARS-CoV-2, including the sites coding the nucleocapsid (miR-29c, miR-29a) and ORF1ab (miR-29a, miR-29b-1, miR-29c-3p, miR-23a) proteins [30]. The latest findings have also presented that neuropilin-1 has a significant role in SARS-CoV-2’s cellular internalization; through merging functional and bioinformatic procedures, one study team defined miR-24 to be a transcriptional modulator of neuropilin-1 [39]. According to very interesting study results, miR-1207-5p can affect the genome of SARS-CoV-2, causing dysregulation of *CSF1*, which can trigger inflammatory reactions in patients with COVID-19, and stimulating epithelial–mesenchymal transition, which may lead to pulmonary fibrosis [22]. Moreover, it has been reported in different studies that hsa-miR-98/23b, through splicing *IFN-β* and *VP1* genes, binds to the SARS-CoV-2 S protein; hsa-miR-497-5p/510-3p/624-5p binds to RNA of SARS-CoV-2’s S glycoprotein; hsa-miR-15b-5p/196a-5p/622/761/A2r/A3r bind to the S gene transcript; and miR-1234-3p/338-3p/4661-3p/4761-5p/4464/7107-5p/885-5p target the S transcript’s receptor-binding site [23,40–42]. Also, the *CLEC4M* gene is additionally associated with S glycoprotein expression and may be inhibited by hsa-miR-5187-5p and hsa-miR-4462 [43]. Furthermore, miR-3613-5p and miR-1307-3p were assumed to suppress viral replication through binding to the 3′ UTR of the viral genome [44]. Several human miRNAs bind to *ORF1ab*, which encodes enzymes of viral translation and replication [45]. *ORF3a* and *ORF1ab* have been stated to be bound by hsa-miR-203b-3p, which inhibits virus replication in the case of influenza A [46]. hsa-miR-101 and hsa-let-7a have been shown to cleave *IFN-β* and *ATP5B* gene transcripts and so target nonstructural protein synthesis of SARS-CoV-2 [40]. hsa-miR-21-3p, hsa-miR-195-5p and hsa-miR-497-5p commonly show expression in epithelial cells in the respiratory tract, possess an elevated target potential against the genome of SARS-CoV-2 and are assumed to be functional against the virus via deletion of the nucleotides in the coding regions of the viral ssRNA [41]. Human miRNAs can inhibit SARS-CoV-2 delivery and attack. The N glycoprotein is a significant antigen of SARS-CoV-2, participating in the packaging of the genome and release of viral particles, and is encoded by *ORF9* [40]. A mixture of antisense miRNAs binds to *ORF9*, 3′ UTR and 5′ UTR parts supposed to suppress viral translation machinery and assembly of particles. hsa-miR-378 and hsa-miR-126 prevent viral N protein production through suppression of translation and splicing of the *IFN-β* gene transcript, respectively [40]. These findings show that these host miRNAs provide resistance against SARS-CoV-2 infection by regulating the SARS-CoV-2 genome.

Garg *et al.* investigated differences in circulating miRNA expression levels between COVID-19 patients, healthy controls and patients with influenza-associated acute respiratory distress syndrome (influenza-ARDS), and discovered significantly increased serum expression levels of proinflammatory miR-155/21/208a and miR-499 in COVID-19 patients compared with healthy subjects [47]. Furthermore, in a validation cohort, they found significant differences in serum miR-155/21/499 levels between COVID-19 and influenza-ARDS patients, indicating that the miRNA profile can be used for detecting and distinguishing these two diseases with high sensitivity [47]. Additionally, miR-208a/499 and miR-155 showed significant overexpression in COVID-19 patients, while miR-21 and miR-126 were present at low levels in the serum of influenza-ARDS patients [47].

In an interesting study using computational analysis of the SARS-CoV-2 genome, the authors revealed a miRNA encoding sequence specific to the SARS-CoV-2 genome and pre-CvmiR-5 sequence that forms CvmiR-5-5p, a mature viral miRNA, which is highly conserved among SARS-CoV-2 strains [48]. According to the computational prediction, neuronal signaling pathways and the development of nervous system genes, neuron development and neuron differentiation have emerged as potential targets of viral CvmiR-5-5p [48], indicating that this may serve in the near future as a predictor of COVID-19-induced neurological symptoms [48].

Experimental data have shown that RNA viruses can produce small miRNA-like RNAs that are the same as the host ones, called human-identical sequences (HISs), which interact with human genome elements regulating different pathways [49,50]. These miRNA-like sequences can repress mRNA expression, thus causing various disease symptoms during the infection. Li *et al.* have shown that HIS, HIS-SARS2-CoV-2, and different noncoding miRNA-like sequences can regulate the expression of both nearby and distant genes [49]. For example, HIS-SARS2 interferes with immune response gene transcripts encoding inflammatory mediators – which was confirmed on fibroblast, kidney and umbilical endothelial cell lines [50,51] – and can influence the COVID-19 outcome through the nuclear activating miRNAs that interact with enhancers of genes such as the gene encoding for hyaluronan, whose accumulation was associated with progression and severe COVID-19 outcome [49]. Antisense oligonucleotides for HIS and degradation induced by Cas13d resulted in reduced hyaluronan levels, indicating that inhibitors of hyaluronan production may serve to prevent severe forms of the disease and that HIS levels may predict disease severity [49].

## miRNAs as potential indicators of worse clinical outcomes in SARS-CoV-2-infected individuals

Eukaryotic miRNAs influence the virus life cycle – replication and promotion – and the propagation of viral infections. The cellular antiviral response might also be significantly altered by host miRNA expression level changes [52]. As an early event, SARS-CoV-2 attacks and destroys T lymphocytes and activates the immune response, which also results in apoptosis of infected cells. The SARS-CoV-2 cell entry is determined by the interaction of viral spike S glycoprotein and ACE2, which serves as a receptor for viral entrance. The reaction is accompanied and catalyzed by TMPRSS2 [53]. During the progress of the disease, the virus particles break through the endothelial barrier, which as a result lowers tissue oxygenation, inducing hypoxia that can lead to numerous complications and death [54].

The question of whether miRNAs can be potential indicators of worse clinical outcomes among SARS-CoV-2-infected individuals should be addressed from two directions. First, the reduction of specific miRNA levels in affected tissue might promote viral replication and trick the host immune response, bearing in mind that miRNAs are master regulators of the immune response [52]. On the other hand, if SARS-CoV-2 infection upregulates some miRNA molecules whose underexpression is necessary for physiological cell processes, then the higher levels of those miRNAs might be used for the prediction of patients’ outcomes. In the cases where SARS-CoV-2 infection downregulates and thus reduces specific miRNA molecules involved in the maintenance of adequate immune response and the prevention of cardiovascular diseases such as stroke or cardiac failure, then it can be said that lower levels of those miRNAs might indicate a potentially worse outcome. SARS-CoV-2’s action in the human body might also be described as a ‘sponge’ for some specific miRNA molecules, via lncRNAs. In some cases, lower levels of some specific miRNAs, sponged by lncRNAs, might be a signal for disease worsening [55].

Although the coronavirus primarily affects the respiratory tract, it can attack a wide range of organs and components of the cardiovascular system, digestive and urinary tracts [56]. miRNAs are associated with cardiovascular diseases, increased coagulopathy, diabetes, acute respiratory syndrome, cytokine response and so on; miRNAs involved in inflammation, the immune response and immune toxicity should be more closely investigated as early biomarkers for various complications and severe COVID-19.

It has been noticed that there are associations between diabetes and obesity and higher rates of hospitalization and mortality, especially among older COVID-19 patients [57]. Also, it has been recently described that patients with hypertension, various coagulopathies and diabetes are more susceptible to developing severe forms of COVID-19 [58]. Lower levels of miR-146a expression have been associated with various conditions as described by various authors [58–60]. Downregulation of miR-146a might be an indicator of COVID-19 severity due to the dysregulated host immune response [59]. Cytokine storm-induced ARDS has destructive effects on the immune system. The cytokine storm might result in a variety of organ failures and can cause death. Drugs (perhaps including miRNA mimics) that target cytokines IL-1, IL-6, IL-18 and IFN-γ might be useful to prevent poor outcome and cytokine storm [61]. One of the drugs targeting IL-6 is tocilizumab. A study by Sabbatinelli *et al.* investigated differences in miR-146a levels between patients responding to this anti-IL-6 receptor drug and those who did not respond [62]. Nonresponders to tocilizumab had significantly lower serum levels of miR-146a after the treatment than responders. Also, patients with the lowest miR-146a levels had the worst outcome and complications, suggesting that a drop in miR-146a levels might predict the adverse outcome of COVID-19 patients [62]; this might be used as an indicator for a change of therapy protocols in those patients in the near future. The second miRNA candidate that accompanies miR-146a in the immune response, but in the opposite direction, is miR-155, a proven regulator of the immune–inflammatory and antiviral response. miR-155 was associated with lung inflammation, the severity of the disease and higher mortality rates within animal models of various respiratory diseases [63–65]. In the study of Soni *et al.* it was shown that miR-155 extracted from nasopharyngeal swabs was significantly elevated within COVID-19 patients compared with COVID-19-negative individuals. Additionally, the same authors showed that anti-miR-155 delivery increased survival rates and improved body weight of transgenic mice and reduced the inflammatory response, indicating that this miRNA is not only a potentially good predictor of severe disease but also a potential target for therapy during severe SARS-CoV-2 infection [65].

Diabetes progression reflects cardiovascular and renal system complications. The presence of diabetes is shown to be associated with a twofold increased chance of getting a severe form of COVID-19 and an adverse outcome [66]. Fulzele *et al.* reviewed and listed several downregulated miRNAs (miR-15a-5p/15b-5p/30b-5p/30e-5p/520c-3p) associated with various pathological conditions such as coronary artery disease, kidney disease, aging, obesity, diabetes and myocardial injury, which had the ability to interact with and target the SARS-CoV-2 genome. According to their review, the downregulation of these miRNAs might indicate a worse disease scenario [67]. Diabetes induces blood vessel damage and increases the chance of thrombosis during COVID-19 infection [68]. Cardioprotective miRNAs such as miR-133a would be expected to be downregulated during SARS-CoV-2 infection, as it has been seen especially associated with older age [67,68]. Kim *et al.* described by bioinformatics analysis that miRNA molecules that have binding sites for three viruses related to severe respiratory complications commonly bind to Middle East respiratory syndrome coronavirus, SARS-CoV and SARS-CoV-2 [32]. miRNA molecules with the highest-ranking scores for binding to SARS-CoV-2 were as follows: miR-15a-5p/15b-5p/16-5p/195-5p/4288/6838-5p. Furthermore, the same authors compared expression levels of miRNAs in hamster lung tissue before and after SARS-CoV-2-induced infection. Among the investigated miRNAs, miR-15b-5p, miR-140-3p and miR-422a were downregulated in SARS-CoV-2-infected hamster lung tissue, while miR-195 and miR-221-3p were upregulated. Among all investigated miRNA molecules, miR-15b-5p and miR-195-5p showed the highest differences between infected and control (healthy) tissue [32], indicating their potential utilization in future SARS-CoV-2 panels for both detection and stratification of patients into different risk groups. Furthermore, the authors found miRNAs that target *ACE2*, such as miR-582-5p/588/587 [32], which also might be considered for future prognostic miRNA panels. In another study, bioinformatic analysis of available experimental data indicated that miR-21-3p was over-represented in the lungs of a mouse during SARS-CoV-2 infection compared with noninfected controls, as well as mmu-miR-21a-5p [35]. In the study by Sabbatinelli *et al.* miR-21-5p and miR-126-3p were shown to be significantly lower in COVID-19 patients compared with healthy individuals, but unlike miR-146a, changes in the expression levels of these two miRNAs did not show any association with response to tocilizumab [62]. miR-18 overexpression might be one of the candidate events preceding SARS-CoV-2-associated nephropathy and plays a significant role in *ACE2* expression silencing [56]. Lower levels of miR-15a-5p were associated with renal disease, while downregulation of miR-520c-3p was associated with diabetes and obesity complications [67]. Candidate miRNAs with the potential to be associated with adverse outcomes are presented in Table 1.

**Table 1.** Candidate microRNA level alterations that might be associated with COVID-19 severity.

miRNA	Direction of changes	Organ/system association	Description	Ref.
miR-146a	Lower levels	Cardiovascular, immune system	Lower levels associated with worse outcome and response to tocilizumab	[58]
miR-155	Higher levels	Cardiovascular, immune system	COVID-19 patients had higher levels compared with COVID-19-negative individuals; anti-miR-155 and reduced inflammatory response and disease severity	[59]
miR-15b-5p/140-3p/422a	Downregulated	Respiratory system	Significantly different between hamster lung tissues with and without infection; bioinformatic analysis of datasets	[32]
miR-195-5p/221-3p	Upregulated	Respiratory system	Significantly different between hamster lung tissues with and without infection; bioinformatic analysis of datasets	[32]
miR-21a-3p/21a-5p	Overexpressed	Respiratory system	Higher expression in infected mouse lung tissue	[34]
miR-21-5p/126-3p/146a	Downregulated	Immune system	Lower in COVID-19 patients compared with healthy individuals	[55]
miR-15a-5p/15b-5p/30b-5p/30e-5p/520c-3p	Downregulated	Cardiovascular system	Coronary artery, kidney disease, aging, obesity, diabetes, myocardial injury	[61]
miR-133a	Downregulated	Cardiovascular system	Cardioprotective; lower levels proposed as indicators of adverse outcome in diabetic patients	[62]
miR-18	Overexpressed	Renal system	Associated with SARS-CoV-2-related nephropathy; silences *ACE2* expression	[50]

According to this review, altered levels of miR-18/15a-5p/15b-5p/133a/140-3p/146a/155/195/21-3p/21a-5p/221-3p/422/520c-3p might be considered to be more deeply investigated and then validated as prognostic biomarkers for early detection of SARS-CoV-2 infection and prediction of a severe form of COVID-19. A panel of miRNA indicators which include key miRNAs altered in the cardiovascular disease spectrum and diabetes and which are involved in immune response could predict the potential development of a severe form of SARS-CoV-2 infection in a timely manner. miRNAs also have the potential to be used for early stratification of patients who might develop severe disease, distinguishing them from those who will have moderate or mild disease. In other words, differences among miRNA levels might be associated with a low or high risk of developing complications. miRNA expression might also be used to guide clinicians to choose the most effective treatment model. We assume that a particular miRNA or set of miRNAs might also be used to predict the type of complications, whether it is a potential cytokine storm, cardiac failure, thrombosis or severe pneumonia. Further detailed investigations are needed to define miRNA signatures that might distinguish specific responses to various infectious diseases, responses to therapy and prognosis.

## miRNAs as potential indicators of better clinical outcomes in SARS-CoV-2-infected individuals

Viral miRNAs, having similar function and structure as human miRNAs, are encoded by the viral genome [17]. miRNAs encoded by the SARS-CoV-2 genome can modulate the inflammatory reaction and immune system of the host in the course of viral infection. For example, MR147-5p binds to *ARRB2* and *CXCL16* enhancers (two inflammation-associated actors), MR385-3p targets the 5′ UTR of *TGFBR3* (a main immune system receptor), MR147-3p binds to the *TMPRSS2* enhancer (a receptor cooperating ACE2, a gate for the entry of virus to the host), MR66-3p acts on the *TNF-α* enhancer (a significant cytokine concerning the cytokine storm), MR328-5p and MR359-5p interact with RARA and MYH9 (two virus infection-associated gene products), respectively, and MR198-3p targets the *ADAR* enhancer (an interferon system reaction-associated protein) [17]. Also, MR147-3p and MR2-5p bind to the apoptosis-associated proteins RAD9A and CHAC1, respectively, which possibly take part in the apoptotic machinery triggered through viral infection-caused disorders of host cells [17]. Therefore, it can be inferred that the downregulation of viral miRNAs MR385-3p/147-5p/66-3p/147-3p/198-3p/359-5p/328-5p/2-5p gives an indication of lower SARS-CoV-2 pathogenicity [50]. Moreover, the gastrointestinal symptoms caused by COVID-19 may be regulated by virus-encoded MR147-3p enabling efficacious invasion of the virus into gut cells, and thus lower MR147-3p expression can be a sign of lower pathogenicity of SARS-CoV-2 [27].

One research team conducted a transcriptomic sequence analysis of both mRNAs and noncoding RNAs in whole blood for six severe and six moderate COVID-19 patients and four healthy controls; they observed that miR-99a-5p, miR-31-5p and miR-181a-2-3p had lower expression levels in severe COVID-19 patients only, compared with the healthy controls. Thus it can be inferred that alterations in these circular miRNAs might serve as biomarkers to determine whether a subject catches COVID-19 and whether the symptoms are severe or moderate [69]. Moreover, several host miRNAs (e.g., hsa-miR-323a-5p, hsa-miR-20b-5p, hsa-miR-17-5p) possess a suppressive function on the inflammation-related pathway and decrease acute lung damage and other organ injuries observed in infection with SARS-CoV-2 [70]. Also, these miRNAs encoded by the host genome may change many host inflammation-related reactions to suppress supplemental injury to sensitive organs like the lungs, preventing the lung against probable damage by targeting TGF-β, VEGF, IGF-1, integrin and PAR1 signaling pathways [70]. Additionally, the p65 subunit of NF-κB is a gene product encoded by the *RELA* gene which regulates apoptosis, inflammatory processes and immune reactions. hsa-miR-6749-3p, hsa-miR-3529-3p and hsa-miR-516b-3p may inhibit the *RELA* gene’s activity via targeting its 3′ UTR and may reduce COVID-19-related symptoms [37]. Furthermore, SARS-CoV-2 is a member of the Coronaviridae family and has been shown to modulate the miRNA repository of the host, therefore managing the virus’s spreading and proliferation. In addition, many different researchers supposed that miRNAs triggered by the coronavirus may modulate antiviral immune reactions of the host such as virus detection, cytokine production and T-cell-regulated cell death. For example, OC43, which is one of the proteins found in the coronavirus nucleocapsid structure, binds to miR-9 and activates NF-κB, a typical proinflammation-related signaling machinery that has been related to various chronic illnesses and virus-associated diseases via its downregulation by the virus invasion [71].

## Conclusion

Through this work, we review evidences showing that miRNAs can possibly provide clinically crucial information about susceptibility to SARS-CoV-2 infection, therapeutic potential against SARS-CoV-2 infection, resistance to SARS-CoV-2 infection, worse clinical outcome in SARS-CoV-2-infected individuals and potential indicators that disease would be less harmful for SARS-CoV-2-infected individuals. We recommend further studies with satisfactory statistical power to precisely evaluate the potential usage of miRNAs for clinical purposes.

## Future perspective

The latest research shows that miRNAs possess the potency to be utilized as therapeutic and diagnostic biomarkers. Thus their detection and confirmation are crucial for developing the infection diagnosis and clinical analysis in COVID-19. Moreover, as expressed above, miRNAs mediate different biological functions and lung disease induced by COVID-19. Thus, by exploring this association, researchers and scientists may utilize miRNAs and miRNA-interacting genes to overcome COVID-19.

**Executive summary**

**miRNAs contributing to susceptibility to SARS-CoV-2 infection**

- The interaction of other molecules and miRNAs in the duration of SARS-COV-2 infection could be an important issue to enable viral entrance to the host.

**miRNAs having therapeutic potential against SARS-CoV-2 infection**

- The miRNAs have been observed to take crucial functions in many regulatory roles affecting the prognosis of viral infection. The miRNAs have a probable function in increasing and decreasing expression of the ACE2 receptors. The clinical applications related with the use of miRNAs to fight the detrimental outcomes of SARS-CoV-2 infection.

**miRNAs contributing to resistance to SARS-CoV-2 infection**

- Host miRNAs can show a well-organized role in suppressing viral attack of the host system through arresting target pathways needed for viral diffusion and other crucial pathways required for virus penetration and replication.

**miRNAs as potential indicators of worse clinical outcomes in SARS-CoV-2-infected individuals**

- miRNAs can be possible indicators of worse clinical consequences among SARS-CoV-2-infected individuals via two directions. First, the reduction of specific miRNA levels in affected tissue may promote viral replication and trick the host immune response. Second, if SARS-CoV-2 infection upregulates some miRNA molecules whose underexpression is necessary for physiological cell processes, then the higher levels of those miRNAs may be used for the prediction of patients’ consequences.

**miRNAs as potential indicators of better clinical outcomes in SARS-CoV-2-infected individuals**

- Even though host- and SARS-CoV-2-derived miRNAs are known to play fundamental roles in the pathophysiological development of COVID-19, their contributions are not sufficient to supply significant and direct support for the therapy and prevention of COVID-19.
- An additional investigation of miRNAs encoded by SARS-CoV-2 is promptly required to analyze their usage for COVID-19 diagnosis, prognosis and therapy.
